# Homology of the Dental Tissues, Their Susceptibility to Suppressive Economy, and Their Present Degeneracy in Man

**Published:** 1877-08

**Authors:** A. H. Thompson

**Affiliations:** Topeka, Kansas


### SELECTIONS.

### THE HOMOLOGY OF THE DENTAL TISSUES, THEIR SUSCEPTIBILITY TO SUPPRESSIVE ECONOMY, AND THEIR PRESENT DEGENERACY IN MAN.

BY A. H. THOMPSON, D. D. S., TOPEKA, KANSAS.

### *Part III. Present Degeneracy in Man.*

The fact of the degeneracy of the tissues of the teeth of contemporaneous man of the current age from a perfect type requires neither demonstration nor reiteration. To every dental surgeon it is a matter of every-day observation in the treatment of dental lesions, and to nearly every member of the human species it is a matter of subjective experience—too often, indeed, made apparent by the acute suffering of which it is the cause, and the inconvenience it occasions by the loss of the teeth.

The query of *why* this should be so is probably as old as history. Defectiveness of the teeth, indeed, antedates written records, although such phenomena of disease as they present in human remains found in pre-historic sepulchres are more infrequent than occur in savage tribes to-day, and are but a fraction of that manifested in civilized nations of our time. There has been progressive increase in diseases of the teeth in man since primitive ages, with more or less fluctuation in the rate of progress, until at the present time the aspect that meets the eyes of the dental profession is appalling and distressing, so extensively disastrous have dental diseases become.

This condition must be largely due to mere deterioration of dental structure, to a defectiveness of tissual organization, and although abnormal oral fluids are an important factor of dental disease (and their deleterious influence is great), it is an important fact that good tooth structure is almost impregnable. Of course dental defectiveness and aggressiveness of the abnormal oral fluids are usually found together, and also good tooth-structure and normal fluids. Yet it is widely observed that teeth of good innate organization rarely succumb to any condition of abnormal salivo that may supervene after their eruption. Most of the apparent exceptions that occur to this rule will be found to be with teeth of defective organization, and the cause of their destruction to have been the same as that of the most apparent case—a difference in degree, but not in kind, of disease. It is fairly demonstrable that the destructive diseases of the teeth of man to-day, and especially caries, are mainly due to defective tissual organization.

In the investigation of the *cause* of this deterioration of dental structure we enter a field filled with controversy, and where truth is yet a stranger. If the cause were once known, it is safe to say that a remedy might be found which, if extensively applied, would bring alleviation of the suffering and inconvenience that have ensued, and confer appreciable benefit upon the race, though it could scarcely cure the evil entirely. But far from this as yet having been the case, or the current being even guided in its course, the destruction progresses with increasing ratio and rapidity. It is within the memory even of a generation yet living, when the general condition of the teeth was much better than is to be found to-day, except in a few individuals, and a century ago scarcely a third of the disease now prevalent existed. The cause of this precipitous deterioration and destruction has baffled all investigators and theorists. It has fallen like a catastrophe, and like a besom of destruction that regards no barrier placed in its path, threatens to sweep the teeth from man within a few centuries, if it should maintain the ratio of increasing speed exhibited during the last hundred years. Whence it has come, why it should be, and whither it tends, are problems which we can only contemplate with a feeling akin to despair, for no human power seems capable of answering.

Contemporary professional thought is almost unanimous in attributing this deplorable condition of affairs to the deficiency of calcium and magnesium salts, particularly the former, in the organic structure of the teeth. These minerals going to harden the dental substance, their deficiency and absence permits solution of the enamel and dentine by acidulated oral fluids, and the disintegration of the entire tooth-body in the disease called caries. The deficiency of these salts is due, it is claimed, to an insufficient supply of foods containing them during intra-uterine existence, infancy, and childhood. The one food which is said to contain the most of these vital mineral constituents is that derived from employing the whole grain of wheat, oats, etc., the outer part of the grain, which is bolted away from the inner part in making fine flour, containing the largest amount of calcium and magnesium salts. This separation of the parts of the grain deprives the human system of the mineral salts, and the teeth are in consequence deficient in inorganic material, and fall a ready victim to caries. It is scarcely too much to say that this theory is to-day the prevalent opinion in the dental profession as to the cause of the present degeneracy of the dental tissues in man; but while there is an appreciable amount of truth in the hypothesis, it has most probably, been carried to an extravagant extreme in the ultra claims that have been made for it.

We feel safe in asserting that, if the people of the United States were compelled to use unbolted flour bread, oatmeal, etc., for ten generations, which would give ample opportunity for the display of the merits of the treatment, the destructive career of caries of the teeth would *not* be stayed. That there would be some effect for the better we willingly concede, for any strict and wholesome regimen would be beneficial, and this one would possess, though in a much smaller degree than is claimed for it, its special merits. The claim that the whole fault of the present degeneracy of the teeth is due to this cause can scarcely be entertained as conclusive. The experiments that have been conducted in the interests of the theory are inconclusive from being too limited and incomplete. Slight benefits have, of course, followed the employment of the regimen reccommended as the one and only remedy, because there is a modicum of merit in it for the purpose, and the food is wholesome and useful. Yet to expect a complete revolution of existing conditions by the method is out of the question.

Special dieting can, of course, bring about particular results in many species of animals, as has been demonstrated, but physiological science has not reached the point where certain results can be promised with mathematical accuracy from the employment of any given kind of food. In this we think the supporters of the unbolted flour theory are at fault; they claim what science has not attained.

The food itself is unfitted for the extensive and usurping employment they would obtain for it, and cannot, for several chemical and physiological reasons, furnish the inconceivably great amount of special nutriment that the teeth require for their resurrection and immortality. On this subject we would notice some observations of Prof. Edward Smith ("Foods") bearing upon this point. "When the bran of wheat is eaten by an animal, whether man or horse, it will be found in the excrement having this layer still perfect, but the more nutritious part, which is covered by the silica, will have been digested in proportion as it was reduced to a fine powder, and allowed to remain sufficiently long in the stomach and bowels. . . . It is of common observation that in proportion as the piece of rind is large and indigestible, so is it a stimulant or irritant of the bowel. So far, therefore, however rich the rind may be in nutritive elements, it is more likely to prevent than to sustain nutrition, since it will lead to the quicker removal from the body not only of itself, but of other and perhaps more nutritious matter. When, however, it is ground into fine powder it does not produce this effect, and although the silicious part may still be undigested, the lignine and starch which it covers may be partially digested and promote nutrition." The grain is composed of different layers, which "increase in nutritive value as we proceed from without inwards. The outside layer, or coarse bran, of the least nutritious, and as the exterior is covered with a layer of silica, it is so far indigestible, and remains as a foreign body in the bowel, setting up irritation, etc. . . . Its nutrative value in this form is limited to the starch and gluten which lie on its inner side. . . . That it can add directly to nutrition is impossible, and whilst it may be very useful to those who are well fed and need a laxative, it may be worse than useless to the ill fed who need nourishment. . . . Good seconds flour is the cheapest and most nutritious, if not the most digestible of the series, and when thirds are produced, or even when the bran is ground to a fine powder and added to the flour, the actual amount of nutrative matter is probably less than with seconds," etc.

We thus see the doubt that is thrown upon this method of dieting for the benefit of the teeth, and yet this is the most promising course of remedy that could be attempted for either prophylactic or reconstructive purposes. The broad truth is that the wave of destruction has gone too far, and that the work of hundreds of generations who have transmitted teeth that became gradually worse and worse cannot be undone by a millennium of dieting. The fault lies deeper than merely being the effect of unfavorable diet, and is the result of those silent but tremenduous forces in nature that make, modify, and destroy species. Some of the multitude of natural laws influencing all animal life in all times and grades of its existence are at work upon the teeth of man, influencing them in a forcible manner for the worse.

Comparative anatomy teaches us that the teeth of man have passed the age and era of their perfection as useful organs, and that they are now, in fact and reality, more or less rudimentary. In their present condition in the species, when many individuals possess dentures of comparative perfection, this fact is not readily apparent, but a close comparison of a number of dentures with that of any other animal whose denture is complete will demonstrate the fact.

The disuse of mastication has been the principal force in reducing the teeth of man and causing them to become rudimentary. Mr. Darwin ("Descent of Man") explains the operation of the law as follows: "Disuse at that period of life when an organ is chiefly used, and this is generally during maturity, together with inheritance at a corresponding period of life, seem to have been the chief agents in causing organs to become rudimentary. The term "disuse" does not relate merely to the lessened action of muscles, but includes a diminished flow of blood to a part or organ from being subjected to fewer alternations of pressure from becoming in any way less habitually active." Every condition of this law is fulfilled in the case of the teeth in man with a precision that is almost an emphasis, and in the passage quoted we find the secret of the rudimentary condition of the teeth in form and structure, and the cause of their deterioration, which promises ultimately to induce their abortion. The effect of disuse as determining and reducing the suppression of the teeth, is paramount to every other force. When a part or organ begins to pass into disuse, as took place in the case of the teeth in man in the troglodytic age, its reduction becomes assured. The diminishing importance of the function of mastication, by reason of the artificial reduction of food then commenced, has continued to increase in direct ratio to the developing of the methods of preparing food. The teeth were less and less actively employed, they began to be superfluous and injurious in a slight and gradually increasing degree, the cost of their maintenance was too great a demand on the system, and not being upheld by direct and active employment, they started on the way to final suppression, which we witness in progress to-day.

The principal force in determining this suppression is *economy of growth* and maintenance. The stimulus of continuous use being slowly withdrawn, the nutrition of the part would gradually diminish, and imperfect development would first supervene as the vanguard of systematic suppression. The part would cease to be normal in structure and form, and this abnormality would increase with continual disuse. Economy of growth would act with increasing force in depriving the part of constructive and sustaining nutriment, and in time the part would be correspondingly defective in structure and form. The force acts in a variety of ways with different tissues, of course, but in the case of the teeth we may now observe what appears to be the attainment of an unusual condition of extreme deterioration. These organs seem to present a remarkable manifestation of this condition of tissues, in being in man at the present time extremely defective in form and structure, so much so as already to appear as rudiments. To this cause must be assigned the result of their special susceptibility to disease. That this is the effect of defective nutrition and the deprivation of formative material there is no doubt; but that it is not the result of any recent want of such material, and that the fault cannot be corrected by special dieting for a few generetions, we feel equally certain. The fault lies in the civilization of the race, and dates from the time the first fire was kindled to cook food, and is as inseparable from civilization as any of its benefits or evils. We must accept the fact of the defectiveness of the teeth, treating and preventing disease by all the means possible, but not indulge in any vain hopes of turning the distroying tide, for the result is inevitable.

But even in view of the preceeding facts it is difficult to perceive how the present precipitous destruction of the teeth by caries can have arisen. Their recent especial susceptibility to the dissolving powers of the oral fluids cannot have been the work of a few generations of mal-nutrition, as the superficial appearances suggest and as many theorists uphold. It seems far more probable that it is the effect of a comparatively sudden deterioration of the oral fluids, which have taken on abnormal action that was easily transmitted and increased. The structure of the teeth has, of course, deteriorated, but it has not been due to the direct and instantaneous effects of mal-nutrition. The cause of the precipitous destruction of these organs during recent generations still remains a mystery, but we think the solution will be found in the principle suggested and the cause in the forces we have mentioned.

Secondary forces are at work assisting in the massacre of the teeth which must also be recognized, and some of which we will briefly notice. What may be called *erratic nutrition* is one of these,—*i. e.*, the irregular nutrition consequent upon underfeeding, high-feeding, and erratic diet. High cooking of all sorts may be included under this head, where highly flavored and prepared dishes to pamper the palate are employed, and which supply both excess and deficiency of nutriment to the system in irregular variability. This kind of food induces indigestion, mal-assimilation, mal-nutrition of the tissues, retardation of growth, and abnormality of gastric and oral fluids; with their attendant train of evils.

*Correlated variation* is a force that has operated upon the teeth and contributed to their reduction,—*i. e.*, the variation of correlated parts as influencing the teeth. In civilized man the extra-vascular parts are subjected to extensive suppression and variability; and abnormality in their condition is manifested also in the teeth. What effect the hairless condition of man may have upon the teeth we cannot trace out, but it has doubtless amounted to an appreciable influence. The correlation between the teeth and the hair and nails of man is most probably more far-reaching and influential than our knowledge or theories suggest at present. We notice that in all animals not posessed of hair, as the classes of fish and reptiles, the enamel is either partially or totally absent; the same may be said of the hairless mammals, as whales, armadillos, etc., though of course it is deficient in some that are possessed of hair. What definite relationship the present defective enamel of the teeth of man may have to his present quasi-hairless condition is difficult to perceive, but there may be an increasing incapacity for the production of corneous tissues in the species, aided by artificial protection of the skin.

*Miscegenation* is a force that, in these days of rapid transit and of migration of peoples, exercises an influence in depressing the integrity of tooth-structure along with other parts of the system. The intercrossing of breeds and races is rarely productive of benefit, when as widely separated as are those cf the human species. Human hybrids are notably deficient in physical completeness and vital power; nutrition is impaired and physiological processes weakened, so that the entire economy is depressed and abnormal. Especially is this true of the American people, who have a continuous stream of foreign and heterogeneous blood pouring into their veins. The depression that has ensued to the physique of the people of the United States may have contributed to the existing extra-defectiveness of the teeth of Americans over those of older nations. The physical type is lowered by the effect of the mixture of races, and will remain so, so long as immigration continues, with the corresponding effect upon the teeth of greater or less manifestation.

*Climatic* and *physical* influences of the soil and air may exercise some effect, especially in America, where but a small part of the people can be considered as approaching acclimatization, in having resided in one locality more than two or three generations. The American people are semi-nomadic, are almost continually on the move and the greatest part of the territory of the United States, *i. e.*, the west, has been settled almost within a generation. Thus the effects of inharmony of climate cannot but act in a depressing manner upon the general system, impairing nutrition and development, and influencing the teeth in a greater or less degree. The same may be said of physical phe nomena, meteorological conditions, etc., and of malaria, etc., from the soil. All these forces figure, with more or less importance and weight, in the drama that is being enacted in the destruction of the teeth in man, and are aided and abetted by others of which we are partially or totally ignorant.

